# The Effects of Gum Acacia on the Composition of the Gut Microbiome and Plasma Levels of Short-Chain Fatty Acids in a Rat Model of Chronic Kidney Disease

**DOI:** 10.3389/fphar.2020.569402

**Published:** 2020-12-22

**Authors:** Maha Al-Asmakh, Muhammad Umar Sohail, Ola Al-Jamal, Banan Mosaad Shoair, Asmaa Yousef Al-Baniali, Salma Bouabidi, Shahd Nasr, Hiba Bawadi

**Affiliations:** ^1^ Department of Biomedical Sciences, College of Health Sciences, QU Health, Qatar University, Doha, Qatar; ^2^ Biomedical Research Center, QU Health, Qatar University, Doha, Qatar; ^3^ Human Nutrition Department, College of Health Sciences, QU Health, Qatar University, Doha, Qatar

**Keywords:** Chronic kidney diseases, adenine, gum acacia, microbiome, short-chain fatty acids, firmicutes, Lactobacillaceae, Proteobacteria

## Abstract

Chronic kidney disease (CKD) may be fatal for its victims and is an important long-term public health problem. The complicated medical procedures and diet restrictions to which patients with CKD are subjected alter the gut microbiome in an adverse manner, favoring over-accumulation of proteolytic bacteria that produce ammonia and other toxic substances. The present study aimed to investigate the effect of GA on 1) the composition of the gut microbiome and 2) on plasma levels of short-chain fatty acids. Male Wister rats were divided into four groups (six each) and treated for 4 weeks based on the following: control, dietary adenine (0.75%, w/w) to induce CKD, GA in the drinking water (15%, w/v), and both adenine and GA. At the end of the treatment period, plasma, urine, and fecal samples were collected for determination of several biochemical indicators of renal function and plasma levels of short-chain fatty acids (SCFAs) as well as characterization of the gut microbiome. Dietary adenine induced the typical signs of CKD, i.e., loss of body weight and impairment of renal function, while GA alleviated these effects. The intestine of the rats with CKD contained an elevated abundance of pathogenic Proteobacteria, Actinobacteria, and Verrucomicrobia but lowered proportions of *Lactobacillaceae* belonging to the Firmicutes phylum. Plasma levels of propionate and butyrate were lowered by dietary adenine and restored by GA. A negative association (Spearman’s *p*-value ≤ 0.01, r ≤ 0.5) was observed between Firmicutes and plasma creatinine, urea, urine N-acetyl-beta-D-glucosaminidase (NAG) and albumin. Phylum Proteobacteria on the other hand was positively associated with these markers while Phylum Bacteroidetes was positively associated with plasma SCFAs. In conclusion, the adverse changes in the composition of the gut microbiome, plasma levels of SCFAs, and biochemical indicators of renal function observed in the rats with CKD induced by dietary adenine were mitigated by GA. These findings are indicative of a link between uremia and the composition of the microbiome in connection with this disease. Dietary administration of GA to patients with CKD may improve their renal function via modulating the composition of their microbiome—a finding that certainly warrants further investigation.

## Introduction

Chronic kidney disease (CKD), a long-term public health problem associated with considerable individual and societal burden, is estimated at present to have a global prevalence of 11–13% (Hill et al., 2016). Moreover, due to the increased prevalence of risk factors for CKD, e.g., obesity, diabetes, and hypertension, as well as our aging worldwide population, the prevalence of this disease is likely to continue rising (Mahmood et al., 2017). In addition, CKD increases the risk for cardiovascular disease and uremia and reduces life expectancy (Ali et al., 2018).

Uremia and the retention of other toxins profoundly modify the barrier function and promote inflammation of the gastrointestinal tract in patients with CKD. Furthermore, both uremia and medical treatment of this condition can change the biochemical and microbial milieu in the gut (Vaziri et al., 2013). In addition, restriction of fruit and vegetable intake to prevent hyperkalemia and administration of antibiotics to treat infections can alter the gut microbiome (Lau et al., 2018; Yang and Tarng, 2018). Eventually, the impaired gut function and microbial dysbiosis in combination can elevate intestinal permeability and promote endotoxemia and systemic inflammation, thereby accelerating the progression of CKD to end stage renal disease (ESRD) (Vaziri, 2012; Hobby et al., 2019).

Vaziri and colleagues (Vaziri et al., 2013), for example, observed a difference in the relative abundance of 175 bacterial taxa in the intestine of rats with CKD and healthy controls; more specifically, fewer bacteria belong to the *Prevotellaceae* and *Lactobacillaceae* families in the disease model. Furthermore, these same authors showed that the guts of patients with ESRD are markedly over-populated with *Enterobacteriaceae*, *Brachybacterium*, and *Pseudomonadaceae*, which express urease, uricase, and enzymes that biosynthesize indole, whereas the numbers of bacteria that can produce short‐chain fatty acids (SCFA) by fermentation of dietary fiber are significantly lowered (Wong et al., 2014). Normally, these toxic nitrogenous products of bacterial fermentation are absorbed through the gut villi, enter the systemic circulation, and are excreted via the urine. In patients with CKD, however, this process is impaired and these metabolites can promote systemic inflammation, toxemia, edema, and hypertension (Evenepoel et al., 2009; Carrero and Stenvinkel, 2010; Gollapudi et al., 2010).

Although controversial, recent evidence suggests that SCFA can protect against kidney damage (Huang et al., 2017), and limited but convincing data indicate that normalization of the gut microbiome through dietary intervention may help prevent systemic toxemia and improve the clinical outcome for patients with CKD (Takayama et al., 2003; Meijers et al., 2010; Nakabayashi et al., 2011; Vaziri, 2012; Vaziri et al., 2013; Sueyoshi et al., 2019). However, these findings remain to be confirmed in larger study cohorts and/or clinical trials.

Previously, it has been shown that gum acacia (GA), a natural product with prebiotic effects (Calame et al., 2008), can reduce oxidative stress and inflammation in the gut of a rat model of CKD (Ali et al., 2010; Ali et al., 2013a). Here, we explored potential effects of GA on the gut microbiome, particularly the SCFA profile, and verify its beneficial influence on kidney function.

## Material and Methods

### Chemicals and Other Materials

A fresh solution of gum acacia (15%, w/v) (SUPERGUM™ EM 10, Lot 101008, 1.1.11, Sanwa-Cho, Toyonaka, Osaka, Japan) was prepared daily. The supplement had a median lethal dose (LD_50_) >16 g/kg for rats. Details about toxicological indexes were published by Ali et al. (2009) (Ali et al., 2009). Adenine (6-Aminopurine, A8626) was purchased from Sigma St. Louis, MO, United States. Kits were used for the determination of: creatinine (Creatinine liquicolor, 10051), albumin (Albumin liquicolor, 10560), urea (Urea liquicolor, 10505) (Human GmbH, Mannheim, Germany), and urinary N-acetyl-β-D-glucosaminidase (NAG; 10875406001) (Sigma St. Louis, MO, United States). The QIAamp DNA Mini Kit 51306 for extraction of genomic DNA (Germantown, MD, United States); and sequencing reagents (Nextera XT Library Prep. Kit, FC-131-1002 and v3 MiSeq kit, MS-102-3003, Illumina, San Diego, CA, United States) were purchased from the sources indicated.

### Animal Housing and Feeding

Male Wister rats (9–10 weeks old, 265 ± 10 g) were housed under standard conditions (22 ± 2°C, 60% relative humidity, and 12-h light-dark cycle with lights on at 6:00 AM) for 7 days before treatment. These animals had *ad libitum* access to tap water and chow containing 25.3% crude protein, 1.12% calcium, 0.85% phosphorus, 0.35% magnesium, and 2.5 IU/g cholecalciferol (Oman Flour Mills, Muscat, Oman). The experimental site was located in Muscat, Oman. The latitude of Muscat, Oman is 23.614328, and the longitude is 58.545284. Muscat, Oman is located at the GPS coordinates of 23° 36′ 51.5808″ N and 58° 32′ 43.0224″ E.

Twenty-four rats were divided randomly into four groups for 4 weeks of treatment as follows:

(1) Group 1, the control group: standard feed and tap water
(2) Group 2, the chronic kidney disease (CKD) group: chow supplemented with adenine (AD) (0.75%, w/w) to induce this condition
(3) Group 3, the gum acacia (GA) group: 15% (w/v) GA in the drinking water
(4) Group 4, the CKD-GA group: administered AD and GA as above

Body weight and intake of both chow and water were monitored once weekly. On the last day of treatment, rats were placed individually in metabolic cages to collect their urine for 24 h.

A total of 24 h after termination of treatment, rats were anesthetized by intraperitoneal injection with ketamine (75 mg/kg) and xylazine (5 mg/kg) and blood (5 mL) and fecal samples were collected. The blood was centrifuged at 900 g at 4°C for 15 min to separate the plasma, which was kept frozen at −80°C until analysis. The fecal samples (collected from three different sites (ileum, cecum, and colon) in each rat were snap-frozen at −80°C and stored until analysis.

This study was approved by the Animal Ethics Committee (Ethical Approval # SQU/AEC/2018-19/01).

### Biochemical Tests

Plasma and urine concentration of albumin, creatinine, urea, and NAG were measured using commercially available kits (Ali et al., 2013b; Al Za'abi et al., 2018).

### Short-Chain Fatty Acids Analysis

SCFAs in rat plasma were derivatized with 3-nitrophenylhydrazine (3-NPH) followed by quantification with liquid chromatography coupled to a triple-quadrupole mass spectrometer—a modification of the procedure described by Han and colleagues (Nielsen et al., 2016). Standard solutions of commercial SCFAs were prepared in 50% acetonitrile at concentrations spanning the range expected in rat plasma (Nielsen et al., 2016). Since plasma not containing SCFAs was not available, these solutions were prepared by dilution with distilled water. The plasma samples were prepared and processed in an identical manner. In connection with derivatization with the isotopically labeled reagent, both the standards and samples were spiked with the same concentration of an SCFA as an internal standard, which allowed determination of recovery. Standard curves were used to determine the concentration of each SCFA.

### Analysis of the Microbiome

DNA was extracted from the fecal samples employing a standard protocol (Ali et al., 2018) and subjected to quality control on the Qubit-4 (Life Technologies, Carlsbad, California, United States) and NanoDrop-2000 (Thermo Fisher Scientific, Waltham Massachusetts, United States) instruments. The V3, V4 hypervariable region of bacterial 16S rRNA gene was amplified by PCR using the broad-spectrum primers (337F/805R) (5′-TCGTCGGCAGCGTCAGATGTGTATAAGAGACAGCCTACGGGNGGCWGCAG -3′

5′-GTCTCGTGGGCTCGGAGATGTGTATAAGAGACAGGACTACHVGGGTATCTAATCC-3′). The 16S rRNA amplicons were then utilized to prepare a library, as described previously (Sohail et al., 2019b; Sohail and Hume, 2019), and these libraries cleaned, normalized, and pooled for sequencing on the Illumina MiSeq platform (San Diego, CA, United States).

### Bioinformatic Analysis

The raw FASTQ data obtained from MiSeq were processed using QIIME2 (Yang and Tarng, 2018), with assessment of the quality of sequencing with the FASTX toolkit. All samples were de-multiplexed and subjected to quality filtration using the DADA2 plug-in. FeatureTable [Frequency] and FeatureData [Sequence] generated from DADA2 were further processed for core matrix analysis and taxonomic annotation (Wajid et al., 2016). The raw FASTQ data has been deposited at NCBI SRA with BioProject accession number PRJNA662560.

### Statistical Analyses

The plasma and urinary levels of metabolites and dietary intake are presented as means ± SEM. The values for the different groups were compared by one-way ANOVA, followed by the Bonferroni test, with *p* values ≤0.05 being considered statistically significant.

The compositions of the microbiomes are presented in terms of the levels of the different phyla and families. Only microbial taxons present at a level of at least 1% in at least one of the four groups were compared statistically. The bacterial compositions are presented as means, and the groups were compared using the non-parametric Kruskal–Wallis test. Furthermore, multiple comparisons of the *p*-values between the study groups were performed using the Benjamini–Hochberg false discovery rate (FDR), with an adjusted *p* ≤ 0.05 being considered statistically significant. Alpha diversity was analyzed using the faith_PD index at a sampling depth of 2,066 and the alpha diversities for the different groups and sites compared with the Kruskal-Wallis pairwise test. Beta diversity is presented as weighted unifrac PCoA plots and contrast between the groups and sites performed using pairwise PERMANOVA. Spearman’s correlation analysis was performed to compare plasma/urine biochemistry and SCFAs with the selected microbial taxa at three different gut sites.

## Results

### Effects of GA on Physiological Parameters

The rats who received adenine in their diet lost approximately 16% of their initial body weight during the experimental period. These rats also consumed more water and urinated more than the controls. Both of these effects were reversed in part by the addition of GA to the adenine-containing diet as well. The relative kidney weight increased approximately three-fold in the CKD than control and GA groups. Fecal output by both the GA and CKD-GA groups was greater than for the other two groups (Table 1).

**TABLE 1:** Effect of gum acacia (GA) on physiological parameters of male rats with chronic kidney disease (CKD) induced by dietary adenine (AD).

Parameters/Treatment	Control	CKD	GA	CKD-GA
Initial bodyweight (g)	265.67 ± 4.59	265.67 ± 7.06	265.83 ± 6.36	265.83 ± 8.10
Final bodyweight (g)	291.17 ± 2.40	222.83^a^ ± 4.96	291.33 ± 7.63	249.50^a^ ^,^ ^b^ ± 8.04
Change in bodyweight (%)	9.71 ± 1.43	−16.02^a^ ± 1.46	9.60 ± 1.26	−6.15^a^ ^,^ ^b^ ± 0.85
Relative kidney weight (%)	0.70 ± 0.02	1.96^a^ ± 0.03	0.72 ± 0.03	1.06^a^ ± 0.06
Water intake (mL)	18.75 ± 0.85	41.20^a^ ± 0.97	21.50 ± 2.18	32.80^a^ ^,^ ^b^ ± 1.51
Urine flow (µL/min)	4.63 ± 0.43	24.31^a^ ± 0.31	5.69 ± 0.56	18.19^a^ ^,^ ^b^ ± 1.09
Food intake (g)	14.02 ± 1.26	10.36^a^ ± 0.95	9.56^a^ ± 0.57	9.46^a^ ± 0.59
Feces output (g)	4.97 ± 0.47	4.76 ± 0.34	8.52^a^ ± 0.69	7.58^a^ ^,^ ^b^ ± 0.60

The values presented are means ± SEM (n = 6).

a Significant difference (*p* < 0.05) of the Control group vs. CKD, GA, and CKD-GA groups.

b Significant difference (*p* < 0.05) of the CKD vs. GA and CKD-GA groups.

### Effect of GA on Renal Function

Administration of adenine in the diet for 4 weeks resulted in significant increases in plasma concentrations of urea and creatinine and urinary NAG (a biomarker of proximal tubular damage), and decreased clearance of creatinine. Proteinuria, an additional indicator of renal injury, was also more pronounced in the rats receiving adenine, an effect that was reduced in part by GA. The values for all the biochemical parameters measured in the GA group were not significantly different from those of control group (Table 2).

**TABLE 2:** Effect of gum acacia (GA) on renal function in male rats with chronic kidney disease (CKD) induced by dietary adenine (AD).

Parameter/Group	Control	CKD	GA	CKD-GA
Plasma creatinine (μmol/L)	16.12 ± 0.48	80.16^a^ ± 5.83	15.76 ± 0.62	58.36^a^ ± 3.75
Plasma urea (mmol/L)	4.66 ± 0.39	42.70^a^ ± 3.92	4.44 ± 0.36	29.02^a^ ± 2.87
Creatinine clearance (mL/min)	1.43 ± 0.11	0.13^a^ ± 0.01	1.99 ± 0.11	0.28^a^ ± 0.02
Urinary NAG (mIU/mL)	45.38 ± 2.84	95.21^a^ ± 1.27	50.47 ± 2.27	71.18^a^ ± 4.63
Urine albumin (mg/L)	7.07 ± 0.34	43.07^a^ ± 3.88	7.77 ± 0.62	29.84^a^ ± 2.23
Urine creatinine (μmol/L)	5,138.80 ± 389.94	426.80^a^ ± 33.86	5,594.40 ± 311.98	887.60^a^ ± 62.41

The values presented are means ± SEM (n = 6).

a Significant difference (*p* < 0.05) of the Control vs. CKD, GA, and CKD-GA groups.

b Significant difference (*p* < 0.05) of the CKD vs. GA and CKD-GA groups.

### Effect of GA on the Microbiome

**FIGURE 1:**
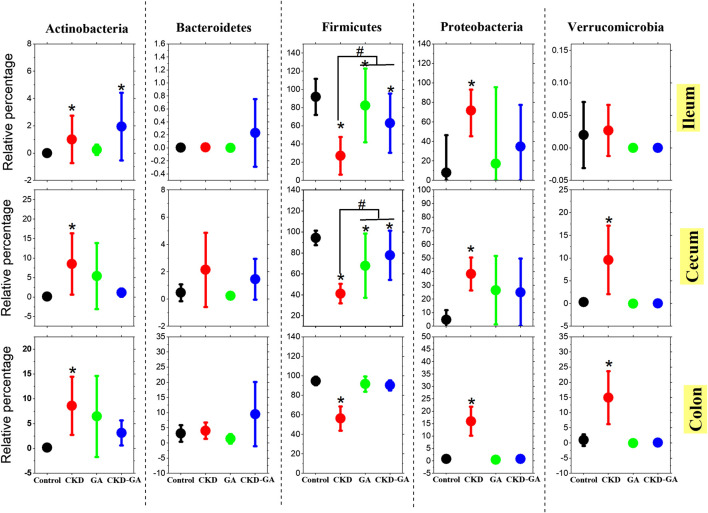
Effect of gum acacia (GA) on the bacterial phyla in the microbiome of male rats with chronic kidney disease (CKD) induced by dietary adenine (AD). The values presented are means ± SEM (n = 6). Phyla that constituted more than 1% of the microbiome in at least one group of animals were analyzed statistically and are presented in this graph. Asterisk (*) represents significant difference (*p* < 0.05) of the Control vs. CKD, GA, and CKD-GA groups. Pound (#) presents significant difference (*p* < 0.05) of the CKD vs. GA and CKD-GA groups.

Firmicutes and Proteobacteria were the most abundant phyla present, with statistically different patterns between the groups of animals. Firmicutes were more abundant in the colon than at the other two locations in the gastrointestinal tract examined (Figure 1). Adenine treatment reduced the level of Firmicutes while elevating the proportions of Proteobacteria Actinobacteria and Verrucomicrobia at all locations. However, supplementation with GA, both alone and in combination with AD, also changed the population densities of these phyla to a certain extent (Figure 1).

**FIGURE 2:**
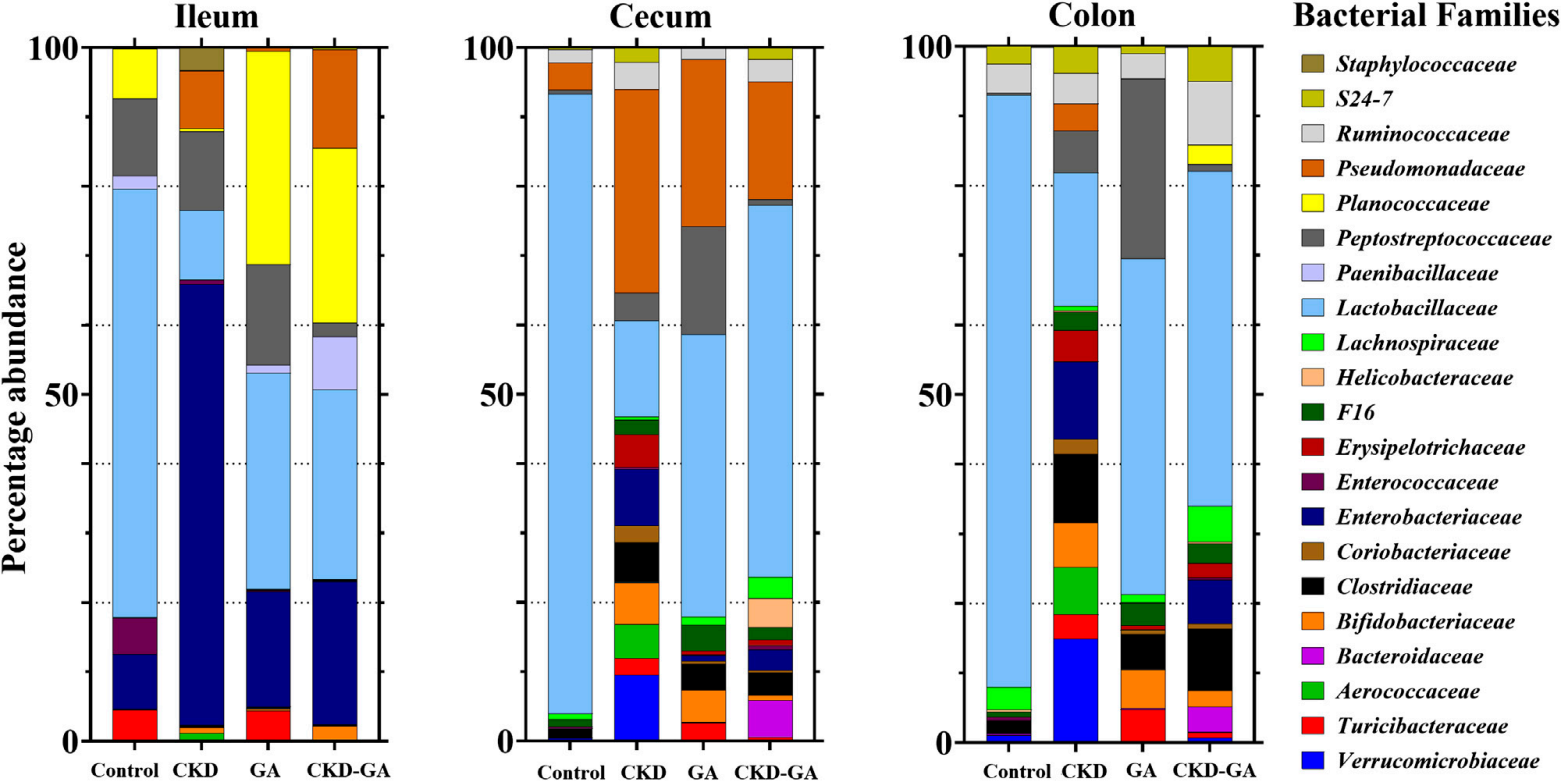
Effect of gum acacia (GA) on bacterial families in the microbiome of male rats with chronic kidney disease (CKD) induced by dietary adenine (AD). The relative abundance of each family at the different locations is represented by the color-coded bar.

At the family level, treatment with adenine reduced the *Lactobacillaceae* count (Table 3; Figure 2), whereas additional supplementation with GA partially reversed this effect at all locations in the intestinal tract. In the ileum, the *Turicibacteraceae* count was lower, while the numbers of *Enterobacteriaceae*, *Pseudomonadaceae*, and *Staphylococcaceae* were higher in the CKD group than control. In the cecum and colon, the *Aerococcaceae*, *Bifidobacteriaceae*, *Clostridiaceae*, *Coriobacteriaceae*, *Enterobacteriaceae*, *Pseudomonadaceae*, *Turicibacteraceae*, and *Verrucomicrobiaceae* counts were highest in the CKD group (Table 3; Figure 2). Dietary administration of GA reduced the abundance of these bacterial families in the CKD-GA group.

**TABLE 3:** Effect of gum acacia (GA) on families of bacteria in the microbiome of male rats with chronic kidney disease (CKD) induced by dietary adenine (AD).

Location and family	Group			
Ileum	Control	CKD	GA	CKD-GA
*Bifidobacteriaceae*	0.00	0.90	0.23	1.89^a^ ^,^ ^b^
*Enterobacteriaceae*	7.88	63.66^a^	16.71	20.68^b^
*Enterococcaceae*	5.22	0.63	0.10	0.11
*Lactobacillaceae*	61.83	9.94^a^	31.21^a^	27.34^a^
*Paenibacillaceae*	1.91	0.06	1.19	7.69^a^ ^,^ ^b^
*Peptostreptococcaceae*	11.14	11.37	14.46	2.02
*Planococcaceae*	7.11	0.45	30.73	25.19
*Pseudomonadaceae*	0.09	8.25^a^	0.51	14.18^,^ ^b^
*Staphylococcaceae*	0.08	3.32^a^	0.04	0.04^b^
*Turicibacteraceae*	4.47	0.21^a^	4.39	0.14^a^
Cecum				
*Aerococcaceae*	0.00	5.10^a^	0.01	0.09
*Bacteroidaceae*	0.04	0.04	0.03	5.35^a^ ^,^ ^b^
*Bifidobacteriaceae*	0.00	6.23^a^	4.91^a^	0.77^b^
*Clostridiaceae*	0.24	2.40^a^	0.52	0.28^b^
*Coriobacteriaceae*	0.12	2.48^a^	0.45	0.36^b^
*Enterobacteriaceae*	0.05	8.47^a^	0.93	3.05
*Erysipelotrichaceae*	0.02	4.88	0.60	0.79
*F16*	1.07	2.19	3.93	1.96
*Helicobacteraceae*	0.02	0.02	0.02	4.22
*Lachnospiraceae*	0.88	0.46	1.19	3.15^a^ ^,^ ^b^
*Lactobacillaceae*	90.24	14.40^a^	41.98^a^	55.41^a^
*Peptostreptococcaceae*	0.54	4.18^a^	16.01^a^	0.84^b^
*Pseudomonadaceae*	4.01	30.38^a^	24.89^a^	17.57^a^ ^,^ ^b^
*Ruminococcaceae*	1.92	4.05	1.71	3.36
*S24_7*	0.26	2.14^a^	0.08	1.78^a^
*Turicibacteraceae*	0.05	2.47^a^	2.71^a^	0.31^b^
*Verrucomicrobiaceae*	0.36	9.85^a^	0.00	0.06^b^
Colon				
*Aerococcaceae*	0.00	7.12^a^	0.01	0.06^b^
*Bacteroidaceae*	0.15	0.07	0.18	3.94
*Bifidobacteriaceae*	0.01	6.66^a^	5.90^a^	2.59^a^
*Clostridiaceae*	0.38	5.31^a^	1.05	0.92
*Coriobacteriaceae*	0.15	2.31^a^	0.61	0.83
*Enterobacteriaceae*	0.01	11.65^a^	0.02	6.71^a^
*Erysipelotrichaceae*	0.04	4.68^a^	0.65	2.25^a^
*F16*	0.74	2.86	3.39	3.14
*Lachnospiraceae*	3.34	0.69	1.26	5.58^a^ ^,^ ^b^
*Lactobacillaceae*	86.22	20.08^a^	50.21^a^	52.23^a^ ^,^ ^b^
*Peptostreptococcaceae*	0.34	6.33^a^	26.95^a^	1.10
*Planococcaceae*	0.00	0.00	0.00	3.21
*Pseudomonadaceae*	0.01	4.06^a^	0.02	0.12^b^
*Ruminococcaceae*	4.19	4.62	3.78	10.09^b^
*S24-7*	2.54	3.97	1.04	5.56
*Turicibacteraceae*	0.07	3.65^a^	4.86^a^	0.83^b^
*Verrucomicrobiaceae*	1.04	15.70^a^	0.00	0.14^b^

The values presented are means (n = 6). Families that constituted more than 1% of the microbiome in at least one group of animals were analyzed statistically and are presented.

The values presented are means ± SEM (n = 6).

a Significant difference (*p* < 0.05) of the Control vs. CKD, GA, and CKD-GA groups.

b Significant difference (*p* < 0.05) of the CKD vs. GA and CKD-GA groups.

**FIGURE 3:**
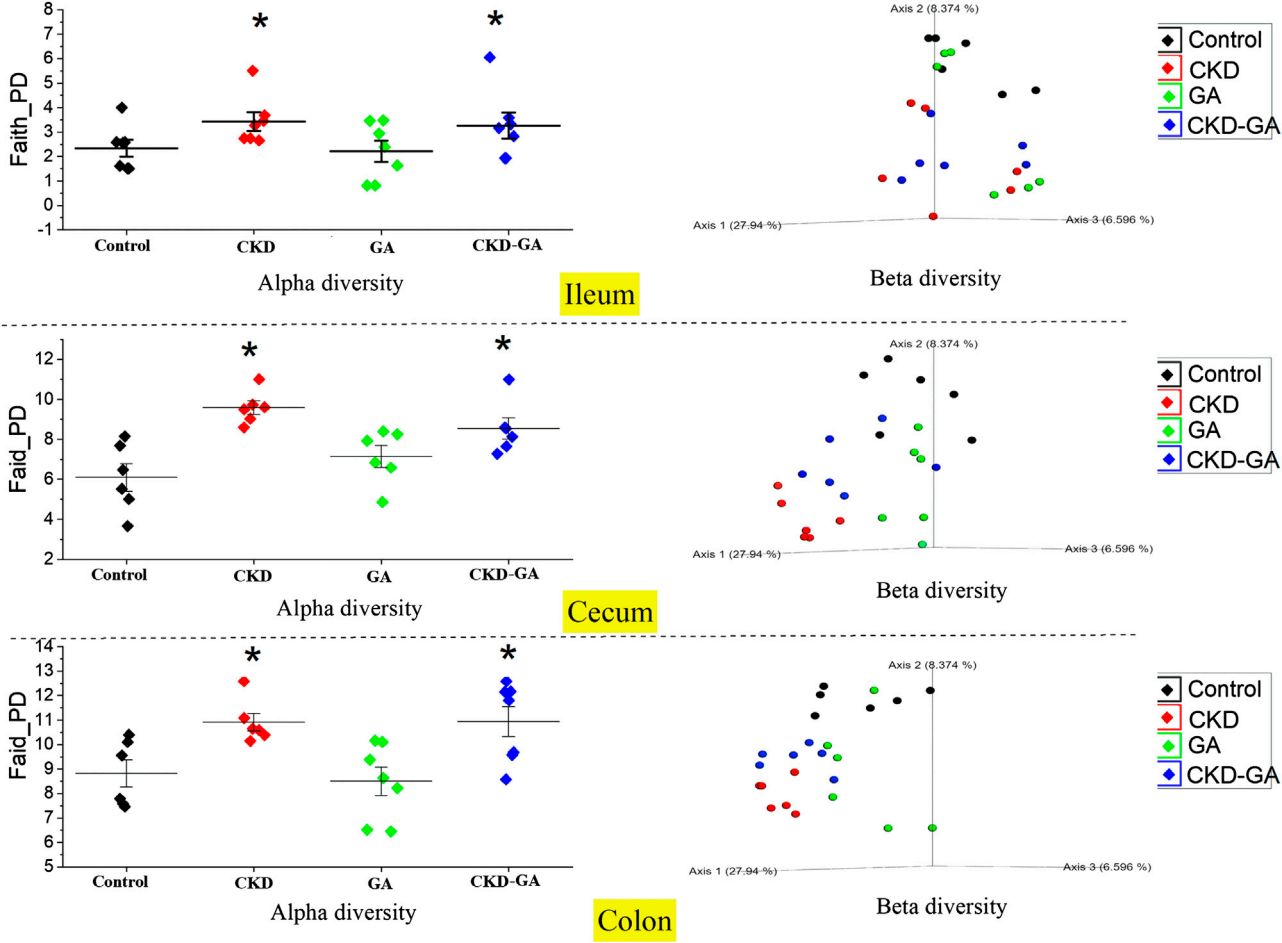
Effect of gum acacia (GA) on the diversity of the microbiome in male rats with chronic kidney disease (CKD) induced by dietary adenine (AD). The alpha and beta diversities of the microbiome at different locations in the intestine are shown. This analysis was performed at a sampling depth of 2,066. Alpha diversity was analyzed using Faith_PD and statistical comparisons carried out with the Kruskal-Wallis pairwise test. Beta diversity was analyzed using weighted_unifrac indexes, expressed as PCoA plots, and compared statistically with PERMANOVA.

At all sites combined, alpha diversity was higher in the CKD than the control and GA groups. However, for the individual locations, this difference was statistically significant in the case of the colon and cecum only. With adenine supplementation, alpha diversity in both the colon and cecum was more pronounced (*p* < 0.01) than in the GA and control groups. Overall, alpha diversity was higher (*p* ≤ 0.01) in the colon, followed by the cecum and ileum in that order. Similarly, the PCoA plots analyzing beta diversity revealed a distinct difference in the clustering patterns in the different groups (*p* = 0.001, Figure 3). Moreover, the pairwise PERMANOVA analysis revealed that clustering at each location and in each group was distinct and differed significantly (*p* ≤ 0.001).

### Effect of GA Supplementation on Plasma Levels of Short Chain Fatty Acids

**FIGURE 4:**
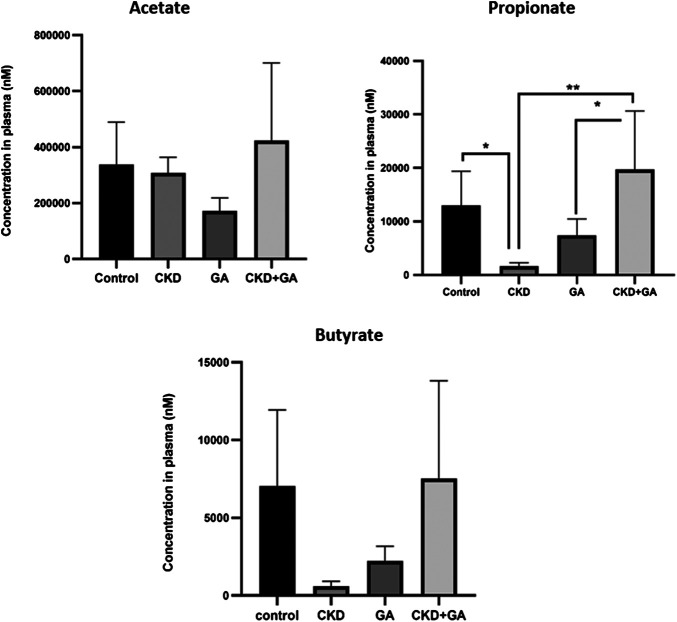
Effect of gum acacia (GA) on plasma levels of short-chain fatty acid in male rats with chronic kidney disease (CKD) induced by dietary adenine (AD). The values presented are means ± SEM (n = 6). *p < 0.05, **p < 0.01, ***p < 0.001 and ****p < 0.0001 as determined by one-way ANOVA with the Dunnett test.

Plasma levels of acetate were the same in all four groups. At the same time, the plasma level of propionate in the rats with CKD was lower than in controls (*p* ≤ 0.05) and additional supplementation with GA reversed this reduction. Although not statistically significant, a similar trend was observed in the case of butyrate (Figure 4).

### Correlation Analysis

**FIGURE 5:**
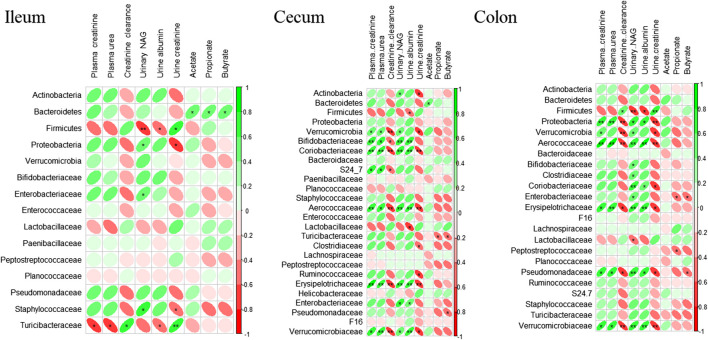
Correlation analysis of rat’s kidney function biomarkers, short-chain fatty acids, and microbiome. Spearman’s correlation analysis was performed using R package “corrplot 0.84” (RStudio version 3.5.0). The color intensity and shape of ellipse indicate the strength of the correlation depicted as r-value. Asterisks in each box indicate the corrected *p*-value; ** ≤ 0.001 and * ≤ 0.01.

The correlation analysis between renal function biomarkers, plasma SCFAs, and individual taxa abundances supports the results obtained from the comparison of the different treatment groups (Figure 5). Association between biomarkers of renal function and microbiome was observed at all gut sites. However, strength of correlation increased as we move further from the ileum to the colon. Phylum Bacteroidetes was positively associated with SCFAs concentrations, albeit the association was strongest in the ileum (*p* ≤ 0.01). Plasma urea, creatinine, urine NAG, and albumin were negatively associated with Firmicutes and positively associated with Proteobacteria and Verrucomicrobia. In contrast, urine creatinine was positively associated with Firmicutes while negatively correlated with Proteobacteria, Verrucomicrobia, Pseudomonadaceae, Erysipelotrichaceae, and Staphylococcaceae. In general, at all three gut sites, all biomarkers of renal function were most closely associated with phylum Proteobacteria, Verrucomicrobia, and Firmicutes and at family level with Aerococcaceae, Verrucomicrobia, Erysipelotrichaceae, and Pseudomonadaceae.

## Discussion

In the present study, we evaluated the effect of GA supplementation on renal function, the plasma profile of SCFA, and gut microbiome of rats with CKD induced by dietary adenine.

Following oral administration, adenine is metabolized to 2,8-dihydroxyadenine, which precipitates and forms tubular crystals that damage the renal tissues (Diwan et al., 2018). Here, dietary administration of adenine caused a loss in body weight; an increase in relative kidney weight, water intake, and urine output; and changes in plasma and urine biochemistry similar to those associated with CKD (Ali et al., 2013b; Ali et al., 2014; Ali et al., 2018), demonstrating the validity of this model. In addition to the biochemical indicators, previous published work validated this model by the morphological and pathological changes of the kidney upon induction of AD (Ali et al., 2014).

GA is used in folk medicine to treat inflammatory conditions as well as clinically to correct uremia in patients with CKD in Middle Eastern countries (Nasir et al., 2012; Ali et al., 2013b). GA provides a source of soluble fibers for gut microbes and enhances fecal nitrogen excretion (Alla and Sadeek, 2018). These effects may explain its anti-inflammatory and anti-oxidative benefits and prebiotic characteristics (Alla and Sadeek, 2018).

In rats with CKD, excessive accumulation of ammonia, derived from the hydrolysis of urea by microbes, promotes the growth of bacteria that express urease (Wong et al., 2014; Vaziri et al., 2016). Our present findings indicate that dietary administration of GA to rats with CKD can improve both their gut microbiome and biochemical parameters.

Here, we observed significant differences in the relative abundances of 62 bacterial clades, in both the alpha and beta indices of diversity, and in the level of two SCFAs between the different study groups. The relative abundance of Firmicutes, for which the main metabolic end-product is butyrate (Macfarlane and Macfarlane, 2003), was lower in the rats with CKD. In addition, many beneficial microbes, such as *Lactobacillus, Coprococcus*, and *Ruminococcaceae*, were depleted in these rats and the plasma levels of their metabolic products propionate and butyrate lowered (Zhang et al., 2010; El Hage et al., 2019). In particular, the proportions of bacterial families belonging to the phyla Firmicutes, Proteobacteria, and Bacteroidetes differed between the groups. Adenine treatment decreased the relative abundances of Firmicutes and increased those of Proteobacteria and Actinobacteria. GA supplementation, alone or in combination with adenine, elevated the relative abundance of Firmicutes and reduced the levels of Proteobacteria.

Proteobacteria is a diverse phylum with a sullied reputation including several well‐known opportunistic pathogens, such as Enterobacteriaceae, Helicobacteraceae, Pasteurellaceae, and Pseudomonadaceae (Sohail et al., 2019a). Vaziri and co-workers (Vaziri et al., 2013) demonstrated elevated numbers of these microbes both in patients with ESDR and the rat model of CKD. This is in line with our observations that a positive association exists between markers of renal function and population densities of Enterobacteriaceae, Pseudomonadaceae, Aerococcaceae, and Verrucomicrobiaceae, which were also significantly more abundant at all intestinal locations in the CKD group—an indication of microbiomal dysbiosis (Sohail et al., 2017; Anwar et al., 2019). Ramezani and colleagues suggested that the uremia associated with CKD promotes rapid influx of undigested proteins into the colon, thereby favoring the proliferation of proteolytic bacteria, microbes that produce phenolic or indolic compounds, and enhance the concentrations of these uremic toxins in the blood (Evenepoel et al., 2009; Mishima et al., 2017). Furthermore, Gryp et al. (Gryp et al., 2020) propose that in rats with CKD *Enterobacteriaceae* and *Verrucomicrobiaceae* express tryptophanase, which is essential for the conversion of tryptophan into indole. A positive correlation in the count of plasma creatinine and urea and gut Proteobacteria may thus explain weak markers of renal function in CKD rats. In contrast, SCFAs, such as propionate and butyrate, produced in connection with the fermentation of fibers, improve renal function by attenuating inflammation, oxidative stress, and apoptosis (Li et al., 2017).

*Lactobacillaceae* of the Firmicutes phylum are well-known for their role in digesting carbohydrates, as well as their probiotic properties (Rossi et al., 2016). Here, the *Lactobacillaceae* counts at the different intestinal locations were significantly reduced in the CKD rats, whereas additional supplementation with GA partially or entirely reversed this change. Numerous studies demonstrated the beneficial effects of dietary supplementation in CKD patients with *Lactobacilli* alone or in combination with prebiotics. (Nakabayashi et al., 2011; Guida et al., 2014; Rossi et al., 2016). These microbes produce butyrate, which enhances the mechanical barrier of the gut and reduces its outflow of uremic toxins (Ramezani and Raj, 2014). Accordingly, the lower abundance of *Lactobacillaceae* CKD rats can explain their higher blood levels of urea and creatinine. Alternatively, these beneficial microbes and GA may act as synbiotics, which are known to improve the composition of the microbiome and, most likely, renal function as well (Alla and Sadeek, 2018).

SCFAs exert multiple beneficial effects on host homeostasis and aid in recovery from disease through their anti-inflammatory, immune-modulatory, and anti-oxidative properties (Puddu et al., 2014; Li et al., 2017; Li et al., 2019). The three major SCFAs acetate, propionate, and butyrate perform important physiological functions: butyrate is the preferential energy source for the gut mucosa; propionate contributes to gluconeogenesis by the liver; and acetate is used in the biosynthesis of cholesterol and fatty acids by the host (Louis and Flint, 2017). In addition, SCFAs have recently been shown to influence multiple aspects of renal physiology and pathology, including inflammation and immunity, fibrosis, blood pressure, and energy metabolism. SCFAs have also been reported to ameliorate kidney injury due to oxidative stress and enhance mitochondrial biogenesis in renal tubular cells (Andrade-Oliveira et al., 2015).

Plasma levels of SCFAs are reduced in patients with CKD (Lustgarten, 2020; Wang et al., 2019) and this reduction may be related to microbial dysbiosis, characterized by drop in Firmicutes and Bacteroidetes, and impairment of the intestinal barrier (Vaziri, 2012; Esgalhado et al., 2017). In the current investigation, the plasma levels of both propionate and butyrate were decreased in rats with CKD induced by adenine. However, supplementation of GA increased propionate concentrations. Similarly, SCFAs concentrations were positively correlated with Bacteroidetes and Firmicutes count, albeit significant association was only observed for Bacteroidetes.

Clearly, SCFAs exert numerous beneficial effects on health. For example, administration of propionate to patients undergoing hemodialysis alleviated their levels of pro-inflammatory parameters, improved insulin resistance and iron metabolism, and was associated with a better quality of life (Marzocco et al., 2018). Butyrate administration to juvenile diabetic rats lowered plasma levels of glucose, creatinine, and urea and attenuated adverse histological alterations (including fibrosis and collagen deposition) in the kidney (Khan and Jena, 2014).

Notably, both animal (Vaziri et al., 2014; Yang et al., 2018) and clinical studies (Krishnamurthy et al., 2012) have highlighted the benefits of a high-fiber diet in improving renal function and reducing inflammation and oxidative stress. In fact, GA supplementation may ameliorate CKD by reducing oxidative stress and inflammation, changing microbiome composition or by inducing an interaction effect of both. However, consumption of a fiber-rich diet increases the risk for hyperkalemia, and patients with CKD are therefore advised to consume lower amounts of fruits and vegetables (Furuland et al., 2018). GA could thus be a good alternative addition to their diet for improving renal function and microbiome composition.

The extrapolation of the findings of this study may be limited to male gender only. Methods used in the current study did not allow for testing impact on microbiome in a mechanistic work. For example, changes in levels of cytokines could have been measured to investigate the link between SCFAs and inflammation.

In conclusion, the observations documented here indicate clearly the importance of the gut microbiome and its metabolites in connection with CKD as well as the ability of supplemental GA to enhance both the growth of beneficial bacteria in the gut and the release of SCFAs that may improve renal function. Several bacterial taxa are associated with renal function and plasma SCFAs. Dietary manipulation of the microbiome in this fashion might provide a novel therapeutic approach to inhibiting the progression of CKD, and further examination of this possibility is certainly warranted. Further work is needed to investigate gut wall integrity, intestinal epithelial barrier function, and quantitative/qualitative alterations of the gut microbiota and their products that associate with the uremia and kidney failure.

## Data Availability

The raw FASTQ data has been deposited at NCBI SRA with BioProject accession number PRJNA662560.
